# Construction of Orthogonal Modular Proteinaceous Nanovaccine Delivery Vectors Based on mSA-Biotin Binding

**DOI:** 10.3390/nano12050734

**Published:** 2022-02-22

**Authors:** Yixin Shi, Chao Pan, Kangfeng Wang, Yan Liu, Yange Sun, Yan Guo, Peng Sun, Jun Wu, Ying Lu, Li Zhu, Hengliang Wang

**Affiliations:** 1 College of Food Science and Technology, Shanghai Ocean University, 999 Hucheng Huan Road, LinGang New City, Shanghai 201306, China; syxsyx0906@163.com; 2 State Key Laboratory of Pathogen and Biosecurity, Beijing Institute of Biotechnology, 20 Dongdajie Street, Fengtai District, Beijing 100071, China; panchaosunny@163.com (C.P.); wangkf1220@126.com (K.W.); liuyanams@163.com (Y.L.); sunyg1995@163.com (Y.S.); yanguobrilliant@163.com (Y.G.); sunpeng990718@163.com (P.S.); junwu1969@163.com (J.W.); 3 College of Life Science, Hebei University, 180 Wusi East Road, Baoding 071002, China

**Keywords:** nanovaccine delivery systems, self-assembled proteinaceous nanoparticle, biotin, mSA

## Abstract

Proteinaceous nanovaccine delivery systems have significantly promoted the development of various high-efficiency vaccines. However, the widely used method of coupling the expression of scaffolds and antigens may result in their structural interference with each other. Monovalent streptavidin (mSA) is a short monomer sequence, which has a strong affinity for biotin. Here, we discuss an orthogonal, modular, and highly versatile self-assembled proteinaceous nanoparticle chassis that facilitates combinations with various antigen cargos by using mSA and biotin to produce nanovaccines. We first improved the yield of these nanoparticles by appending a short sugar chain on their surfaces in a constructed host strain. After confirming the strong ability to induce both Th1- and Th2-mediated immune responses based on the plasma cytokine spectrum from immunized mice, we further verified the binding ability of biotinylated nanoparticles to mSA-antigens. These results demonstrate that our biotinylated nanoparticle chassis could load both protein and polysaccharide antigens containing mSA at a high affinity. Our approach thus offers an attractive technology for combining nanoparticles and antigen cargos to generate various high-performance nanovaccines. In particular, the designed mSA connector (mSA containing glycosylation modification sequences) could couple with polysaccharide antigens, providing a new attractive strategy to prepare nanoscale conjugate vaccines.

## 1. Introduction

In recent years, the development of vaccinology as a field has greatly promoted the novel design of vaccines [1,2]. In particular, research into nanovaccine delivery systems in immunology has continuously provided remarkable insights into the nature of the immune response and has gradually become an area of intense research. In its own right, efficient nanoscale delivery carriers usually have many advantages, such as antigen delivery, depot effects, repetitive antigen display, and cross-presentation, and there are many innovative projects seeking to develop more efficient, adjuvant-free prophylactic nanovaccines [3,4,5,6,7]. Therefore, nanotechnology has had a far-reaching impact on vaccinology and promoted the development of newer classes of vaccines.

At present, a variety of nanoscale materials, such as polymeric particles, self-assembled protein particles, liposomes, and outer membrane vesicles, have been deployed in prophylactic vaccine design [4]. Among these, proteinaceous delivery vectors, such as virus-like particles, self-assembling nanoparticles, ferritin, and encapsulation [8,9,10,11,12,13,14,15,16] have obvious potential advantages, including higher biocompatibility and safety. Our previous study described a fully protein-based, self-assembling, stable nanovaccine to realize the displaying of versatile antigens on it, especially glycan antigens with complex structures [17]. Through the co-expression of glycosyltransferase PglL (a very potent tool enzyme) [18,19,20,21,22], a fusion protein containing the cholera toxin B subunit (CTB) and a trimer forming peptide (CTBTri), polysaccharide antigens could be connected to these particles, self-assembled by CTBTri monomers, under the catalyzing activity of PglL in host cells. In an efficient lymph node draining after vaccination, the antigens bound by these nanovaccines were well presented by antigen-presenting cells for T cell activation and strong prophylactic effects against infection were observed in mouse and monkey experiments. However, similar to most reported proteinaceous delivery vectors, the strategy of simultaneous expression of an antigen and a vector (such as fusion expression) requires the expression of nanoparticles each time when preparing different vaccines, which may affect the self-assembly and yield of nanovaccines due to different antigen structures. Thus, the next step in the development of nanovaccines is how to further realize the independence and universality of nanoparticles.

At present, there have been many coupling technologies to achieve the efficient loading of antigen cargos on delivery carries [23,24]. Although streptavidin—which can bind biotin with high affinity—also has the potential to be a connector between delivery carriers and antigens, the large molecular weight of streptavidin (over 60 kDa) and its obligate tetramer formation for binding to biotin limits its application. Park et al. reported an engineered streptavidin monomer (mSA) with only about 14 kDa that consisted of part of the sequences of streptavidin and rhizavidin and could bind to biotin with high affinity (even reaching 2.8 nM) [25,26,27]. Moreover, the applications of mSA have been deeply explored, and it has been used in fields, such as cellular import, cell surface engineering, antigen loading, and tumor targeting [28,29,30,31,32,33,34,35,36].

Therefore, we envisioned that mSA could function as a connector by fusing with protein antigens or glycosylation site sequences (for loading polysaccharide antigens), so as to couple with biotinylated high-performance nanoparticles, which we have reported on previously. Specifically, we constructed an orthogonal, modular, and highly versatile nanovaccine delivery vector that facilitates combinations with antigen cargos to produce nanovaccines. By the analysis of the cytokine spectrum in the plasma of immunized mice, we found that these nanoparticles could enhance both Th1- and Th2-mediated immune responses. Subsequent binding analysis showed that these biotinylated nanoparticles could interact with both protein and polysaccharide antigens at a high affinity. Our approach thus offers an attractive tool for combining nanoparticles and antigen cargos to generate various high-performance nanovaccines. In particular, the coupling with polysaccharide antigens provides a novel efficient strategy for preparing nanoscale conjugate vaccines.

## 2. Materials and Methods

### 2.1. Construction of rfc and waaI Double Mutant S. flexneri 2a 301

A *rfc* and *waaI* double mutant *S. flexneri* 2a 301 strain, named 301DRW, was constructed using the λ-Red recombination system as described before [37]. Based on the sequences of *rfc* and *waaI* from the whole genome sequence of *S. flexneri* 2a 301 (NC_004337) obtained from the NCBI database, primers were designed using software Primer Primier(6.0, Premier Biosoft International, Palo Alto, CA, USA)and are listed in the Supplementary Materials, Table S1. The upstream and downstream homologous arms (about 500 bp) of *rfc* were amplified from genomic DNA using primers rfc-up-f/r and rfc-down-f/r, respectively. These two fragments were then inserted into pET-kan, harboring a kanamycin resistance gene flanked by FRT sites, creating the plasmid pET-rfcUp-kan-rfcDown. The primers rfc-up-f and rfc-down-r were used to amplify this targeting DNA fragment from pET-Up-kan-Down, and the products were introduced into *S. flexneri* 2a 301/pKOBEG competent cells by electroporation. After being incubated at 30 °C overnight, the colonies growing kanamycin and chloramphenicol plates were identified by PCR using the primers rfc-out-f/r, rfc-in-f/r and kan-in-f/r. Plasmid pKOBEG in each correct clone was cured by culturing at 42 °C overnight, and the resulting strain was named 301DR::Kan. After that, the temperature-sensitive plasmid pCP20 was transferred into 301DR::Kan competent cells to remove kanamycin resistance and then this was cured by culturing at 42 °C overnight, yielding strain of 301DR. The gene *waaI* was knocked out using the same methods and the upstream and downstream homologous arms were amplified by primers waaI-up-f/r and waaI-down-f/r, respectively.

### 2.2. Lipopolysaccharide (LPS) Preparation

Strains were cultured in LB medium at 37 °C overnight and collected by centrifugation at 8000× *g* for 10 min. After being washed three times with ddH_2_O, the cells were resuspended in 3 mL of ddH_2_O per gram of wet weight. After three rounds of freezing and thawing, 90% phenol in equal volume was added and cultures were vigorously shaken at 68 °C for 30 min. Then, the aqueous phase was collected through centrifugation at 8000× *g* for 20 min and an equal volume of ddH_2_O was added again for re-extraction. Residual phenol in the water phase was removed by dialysis. Next, protease K was added, and the extract was incubated at 60 °C for 1 h. After that, the samples were boiled in water for 10 min to obtain LPS.

### 2.3. Silver Staining

LPSs, mixed with an equal volume of 2 × SDS loading buffer, were boiled in a water bath for 10 min and were then separated by SDS-PAGE. Each gel was fixed by immersing in a fixing solution (100 mL containing 40% ethanol and 5% acetic acid) and slowly shaken for 15 min twice. Subsequently, each gel was placed in sensitizing solution (100 mL containing 7 g sodium acetate, 0.2 g sodium thiosulfate, 30 mL ethanol, and 0.25 g glutaraldehyde) and incubated for 30 min. After washing with ddH_2_O three times, the gel was then stained with 100 mL of silver nitrate solution containing 2.5 g silver nitrate and 40 µL formaldehyde. Next, the gel was washed twice (1 min each time) with ddH_2_O and was finally incubated in developer solution (100 mL containing 0.75 g sodium carbonate, 2.8 µL 5% sodium thiosulfate, and 40 µL formaldehyde). Depending on the degree of color development, the reaction was terminated by adding a termination solution (100 mL containing 1.46 g EDTA·2H_2_O). Finally, each gel was washed with ddH_2_O 3 times for 5 min each time.

### 2.4. Protein Expression and Purification

The strain 301DRW harboring pET28a-pglL-CTBTri was cultured at 37 °C until the OD_600_ reached 0.6–0.8, and then induced with 1 mM IPTG at 30 °C overnight. Cells were collected by centrifugation at 8000× *g* for 10 min. The cell pellet was resuspended in buffer A1 (20 mM Tris-HCl, pH 7.5, 10 mM imidazole, and 500 mM NaCl) and was homogenized to release protein. After centrifugation at 8000× *g* for 10 min, the supernatant was loaded onto an Ni Sepharose excel column (GE Healthcare, Piscataway, NJ, USA). Impurities were removed with 5% buffer B1 (20 mM Tris-HCl, pH 7.5, 500 mM imidazole, and 500 mM NaCl), and samples were eluted with 100% buffer B1. Afterwards, the samples were separated using a Superdex 200 column (GE Healthcare) and fractions were analyzed by SDS-PAGE.

### 2.5. Western Blotting

Proteins were separated by SDS-PAGE and Western blotting was performed as described previously [18,19]. HRP-conjugated 6 × His-tag antibody (Abmart, Shanghai, China) was used to detect His-tagged proteins. SARS-CoV-2 (2019-nCoV) spike RBD antibody (Sino Biological, Beijing, China) was used to detect RBD. *S. flexneri* 2a OPS-specific serum (Denka Seiken, Tokyo, Japan) was used to detect polysaccharide antigen of *S. flexneri* 2a 301. HRP-conjugated streptavidin (Solarbio, Beijing, China) was used to detect biotinylated proteins. HRP-conjugated anti-rabbit IgG (H+L) (Biodragon, Suzhou, China) was used as a secondary antibody.

### 2.6. Mouse Immunization

The female Balb/c mice (SPF) used in this work were purchased from Beijing Vital River Laboratory Animal Technology Co., Ltd (Beijing, China). All animal experiments and procedures were performed in accordance with the guidelines of the Academy of Military Medical Sciences Institutional Animal Care and Use Committee (Ethics Approval Code IACUC-DWZX-2021-073). The mice were randomly divided into four groups: PBS, OPS, NP-OPS, and NP-RU. Except for the PBS group, the other groups were immunized with different treatments containing polysaccharides at 2.5 μg/mouse. All groups were immunized 3 times subcutaneously at an interval of 2 weeks. One week after the last immunization, the tail blood was taken, and the serum was separated for further analysis.

### 2.7. Biotinylation and Connection

Nanoparticles were biotinylated by using EZ-Link NHS-LC-LC-Biotin Reagents (Thermo Fisher Scientific, Waltham, MA, USA). Briefly, a 10 mM solution of the biotin reagent that contained 2.0 mg of NHS-LC-LC-Biotin in 350 µL of dimethylsulfoxide (DMSO) was first prepared. Then, 46.2 µL of the 10 mM biotin reagent was added to 1 mL of a 0.5 mg/mL NP-RU protein solution. After incubating at 25 °C for 1 h, biotinylated nanoparticles were produced by removing unbound biotin via dialysis. For antigen loading, these biotinylated nanoparticles were then incubated with mSA-antigen at 37 °C for 1 h.

### 2.8. Enzyme-Linked Immunosorbent Assay (ELISA)

We coated 96-well plates with LPS from *S. flexneri* 2a 301 (10 μg/well) overnight at 4 °C. The next day, each well was washed three times with PBST (PBS with 0.05% Tween 20) using an automated plate washer (ELx50 Washer, BioTek Instruments, Inc., Winooski, VT, USA). After washing and drying, the wells were loaded with 200 μL of blocking buffer (5% skim milk powder in PBST) and incubated at 37 °C for 2 h. Next, the wells were incubated with diluted serum at 37 °C for 1 h. After another washing and drying step, 100 μL of HRP-conjugated donkey anti-mouse IgG (Abcam, China) was added to each well, and the plates were incubated at 37 °C for 1 h. Plates were again washed and dried, and then the Soluble TMB kit (CWBio, Beijing, China) was used for color development and the reactions were stopped with 50 μL of stop solution per well. The absorbance at a wavelength of 450 nm was then measured.

### 2.9. Statistical Analysis

Statistical analyses were conducted using GraphPad Prism version 8.0 (GraphPad, San Diego, CA, USA). Data are expressed as the mean ± SD. A Student’s *t*-test was used for comparisons between two groups. Differences with a *p*-value of < 0.05 were considered significant.

## 3. Results and Discussion

### 3.1. Establishment of Host Cells for Nanoparticle Expression by Knocking Out rfc and waaI in Shigella flexneri 301

**Figure 1:**
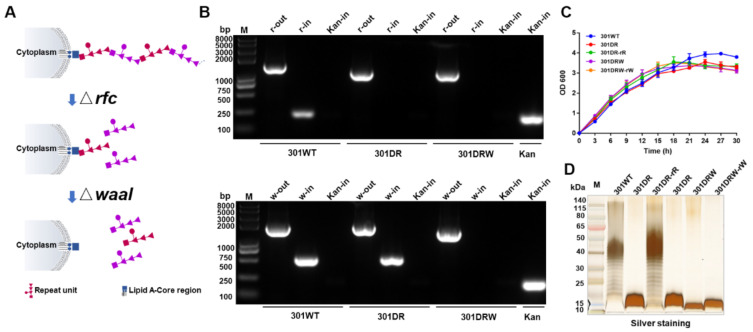
Establishment of *rfc* and *waaI* deletion mutants. (**A**) Schematic diagram of the effect of gene knockout on polysaccharides. *rfc* is an O-antigen polymerase and the knocking out of *rfc* prevents RUs polymerization, resulting in only one RU in the lipid A-core. *waaI* is an O-antigen ligase. The further knocking out of *waaI* prevented the polymerized polysaccharide chain from connecting to the lipid A-core. (**B**) PCR analysis of mutants in the *rfc* and *waaI* genes. (**C**) Growth curve analysis of knockout mutant strains. 301DR-rR indicates a 301DR revertant strain (301DR containing pACY184-rfc), 301DRW-rW indicates a 301DRW revertant strain (301DRW containing pACY184-waaI). (**D**) Silver staining of LPS from various strains. The typical ladder could not be detected by silver staining after *rfc* deletion. 301DR-rR and 301DRW-rW indicated the LPS, extracted from 301DR-rR and 301DRW-rW revertant strains, respectively.

Our previous results have shown that antigens, co-expressed with CTBTri, could self-assemble into nanoparticles in cells, and could induce strong humoral and cellular immune responses by promoting antigen lymph node drainage, endocytosis, presentation, and the proliferation and differentiation of downstream immune cells [17]. To further expand the application of this nanocarrier, we envisaged the construction of independent universal protein nanoparticles, which could be used as a chassis to realize the modular design of antigen and nanocarrier as needed. We started our research by solving the problem of the low yield of expressing individual nanoparticles. Considering the high hydrophilicity of polysaccharides, we intended to embellish the surface of nanoparticles with hydrophilic sugar chains, which could promote protein soluble expression and increase production. Although coupling polysaccharides with protein is a feasible method to prepare proteinaceous nano delivery carriers, the long polysaccharide chains of pathogenic bacteria could elicit polysaccharides specific immune responses. Thus, to avoid this additional burden of an immune response, we constructed a *Shigella* host bacteria that was able to modify the short sugar chain on expressed nanoparticles (Figure 1A). We first knocked out the O-antigen polymerase gene *rfc* [38], which controls the polymerization of polysaccharide repeat units (RU) (containing five monosaccharides) of O-polysaccharide (OPS) in LPS (Supplementary Materials Figure S1), from the *Shigella flexneri* 301 strain using the λ-Red recombination system (Supplementary Materials Figure S2) [37]. This deletion (named 301DR) resulted in only one RU attaching to the lipid A-core of LPS (Figure 1A). On the basis of strain 301DR, we further knocked out the gene *waaI* to block the connection of RU to the lipid A-core, resulting in the novel strain we named 301DRW (Figure 1A and Supplementary Materials Figure S3). The PCR results showed that 301DRW, as a template, was amplified by *rfc* external primers (r-out), and the amplified band was lower than that from the 301 wild type and 301DW but consistent with the 301DR band. Similarly, these amplifications were performed using *waaI* external primers (w-out), and the band was lower than the 301 wild type and 301DR bands but consistent with the 301DW band. No positive bands were amplified by two pairs of internal primers (r-in and w-in) (Figure 1B). These results indicated that we successfully constructed double deficient strains at the gene level. In addition, using strain 301 as a control, by monitoring the growth of different deletion strains over time, we found that the growth rates of these strains were not affected by these gene knockouts, whether as a single deletion of *rfc* or the simultaneous deletion of *rfc* and *waaI* (Figure 1C). In order to further verify the phenotypic changes to polysaccharides, lipopolysaccharides (LPS) from each strain were analyzed by silver staining after SDS-PAGE. The results showed that the typical LPS ladder could not be detected in the 301DR strain due to the deletion of the *rfc* gene. The further deletion of the O-antigen ligase gene *waaI* made the first RU unable to connect to the lipid A-core, and the band of the 301DRW strain in this assay moved lower compared to the 301DR strain (Figure 1D). The above results indicated that we successfully constructed a host, *Shigella*, expressing free RU.

### 3.2. Expression and Purification of Nanoparticles Modified with a Short Sugar Chain

**Figure 2:**
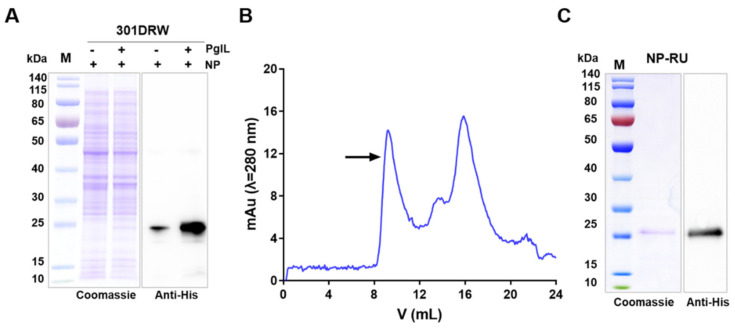
Expression and purification of NP–RU. (**A**) The plasmids pET28a–CTBTri and pET28a–PglL–CTBTri were transferred into strain 301DRW, respectively, and Coomassie blue staining and Western blot assays were performed to analyze the expression of CTBTri (NP) expressed alone or co–expressed with. (**B**) Analysis of purified NP–RU by size–exclusion chromatography (Superdex 200, GE Healthcare). The peak position of NP–RU is indicated by an arrow. (**C**) The purified NP–RU was detected by Coomassie blue staining and Western blotting.

After successfully knocking out the *rfc* and *waaI* genes and verifying their phenotypes, we further determined whether the RU coupling with the nanoparticle increased its expression. Plasmids co-expressing inducible PglL and CTBTri (pET28a-pglL-CTBTri) and expressing an inducible CTBTri (pET28a-CTBTri) were introduced into strain 301DRW. As expected, RU could be coupled to carrier proteins under the catalysis of PglL, and Western blot results show the significantly increased expression of protein in the IPTG-induced 301DRW strain co-expressing PglL and CTBTri (Figure 2A). Furthermore, the protein was purified by affinity and size-exclusion chromatography, and the target protein was eluted in 8–10mL from a column with a 24 mL column volume (Superdex 200 Increase 10/300 GL) (Figure 2B), indicating that the protein existed in the form of polymer. The monomer’s molecular weight was only about 25 kDa, as determined by Coomassie blue staining and Western blotting (Figure 2C).

### 3.3. Characterization of Nanoparticles Modified with Short Sugar Chains

**Figure 3:**
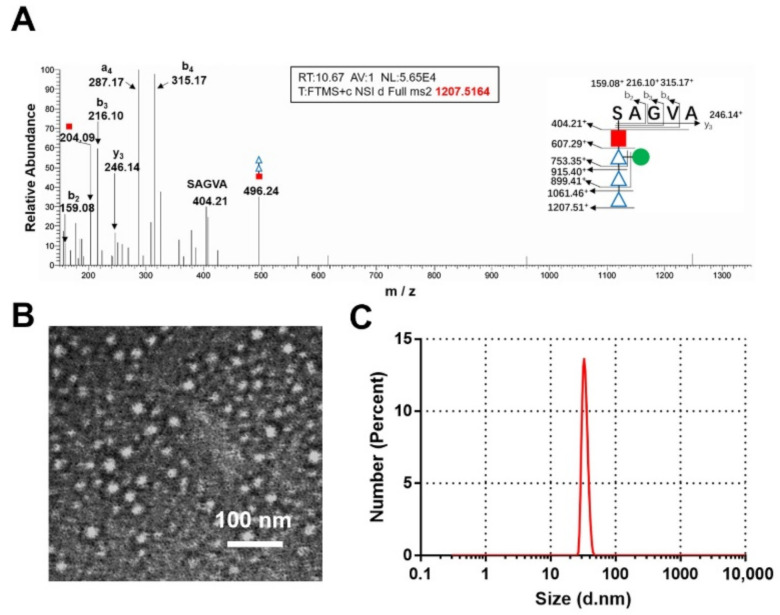
Characterization of NP-RU. (**A**) Tandem mass spectrometry (MS/MS) analysis of NP-RU. MS/MS spectrum of singly charged ions at *m*/*z* 1207.51^+^ contained the pentapeptide SAGVA (*m*/*z* 404.21^+^) and a peptide linked with sugars. The peptide and glycopeptide fragment ions were identified (shown in the inset). (**B**) TEM images of NP-RU. (**C**) DLS analysis of NP-RU.

To confirm RU was successfully connected to nanoparticles, particles were digested with protease K and the glycopeptides were examined by liquid chromatography-tandem mass spectrometry (LC-MS/MS), as described previously [19]. The results showed that singly charged ions at *m*/*z* 1207.52^+^ contained the pentapeptide SAGVA (*m*/*z* 404.21^+^) and one RU (803.3 Da) in the MS/MS spectrum (Figure 3A), suggesting that RU was successfully linked to the glycosylation Ser sites of these nanoparticles. Furthermore, transmission electron microscopy (TEM) analysis of purified extracts revealed a nanoparticle of about 20–30 nm in size (Figure 3B), a finding that was in line with our size-exclusion chromatography results. Dynamic light scattering (DLS) results also revealed a homogeneous size distribution, which was consistent with our TEM results (Figure 3C), in addition to evaluating the stability of this nanoparticle. After being incubated at 37 °C for 48 h, these nanoparticles still showed a good colloidal state and were still monodisperses with a stable size. (Supplementary Materials Figure S4). Thus, the introduction of short sugars did not affect the self-assembly of these proteins, and we had realized a markedly improved expression.

### 3.4. Activation of Immune Responses by Nanoparticles

**Figure 4:**
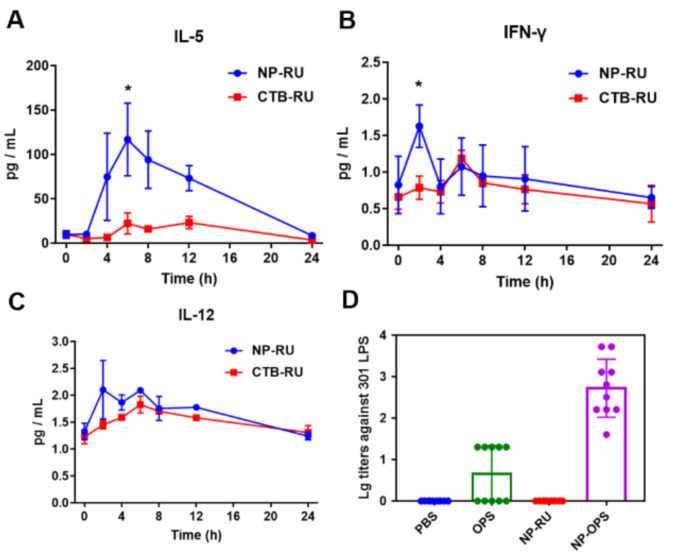
Analysis of the immune activation ability of NP-RU. Where 2 μg of each sample was injected subcutaneously into mice and the plasma at different time points was collected from each mouse. The levels of 12 different cytokines were detected using the liquichip method and the release of the Th2-skewed cytokines IL-5 (**A**) and the Th1-skewed cytokines IFN-γ (**B**) and IL-12 (**C**) had clear differences. Data are presented as the mean ± s.d. A Student’s *t*-test was used for comparison between two groups: * *p* < 0.05. (**D**) Immunogen analysis of RU by injecting 2.5 μg of sugar into each group (except PBS), on days 1, 15, and 29. ELISA was performed to detect antibodies against *S. flexneri* 2a 301 LPS in the serum collected on day 39 (10 days after the last immunization).

Having established that these proteins containing a short sugar chain could be expressed in cells and could assemble into extractable nanoparticles, we next confirmed the immune activation ability of these nanoparticles. After subcutaneous injection of 2 μg of nanoparticles in each mouse, we measured 12 different cytokines in the plasma at different times using the liquichip method. At the same time, an injection of CTB-RU (CTB connected with a single RU) was performed as a control. The results showed that during the 12 h monitoring period, IL-5 was significantly higher than CTB-RU from 4–12 h, even as high as 5-fold higher at 6 h (Figure 4A). As is well-known, IL-5 is mainly secreted by Th2 cells in mice and can promote the maturation of B cells [39]. The increase in IL-5 suggested that these NP-RU could enhance the activation of humoral immune responses. In addition, IL-12 and IFN-γ, indicators of cellular immune responses [40], were also higher in NP-RU injected mice than in the CTB-RU group, although IL-12 was not statistically significant at the peak (Figure 4B,C). Additionally, we found an increase in IL-22, which functions to promote innate immunity [41], after NP-RU immunization (Supplementary Materials Figure S5). This suggested that our nanocarrier could activate the immune system. It has been well-established that the immune response achieved for a particular vaccine agent is strongly influenced by the duration over which the antigen persists, as well as by the antigen’s (size-related) ability to accumulate in lymph nodes [42]. Generally speaking, 15–100 nm is the optimal size for vaccines for direct homing to the draining lymph nodes [43]. In our design, these nanovaccines were about 20–30 nm in size (Figure 3B), which was conducive to lymph node targeting and ensured effective antigen accumulation in the draining lymph nodes. Traditionally, exogenous antigens using CTB as adjuvants or for fusion have been shown to be internalized by antigen-presenting cells (APCs) and further presented in the MHC-II pathway for a humoral response, which mainly requires the assistance of Th2 cytokines [20,44,45,46,47]. Some researchers found that CTB-based vaccines have the capacity to induce Th1 immune responses [48,49,50,51]. Such a specific performance of CTB-based vaccines relative to other vaccines can be attributed to their different formulations. Once a CTB-based vaccine has been formulated at the nanoscale size, the lymph node accumulation, APC uptake, and MHC-I cross-presentation can be elevated [17,43,52,53]. These enhancements allow APCs to secrete Th1 cytokines to facilitate cellular responses. In addition, dendritic cell activation strategies [44] and administration routes [48,54] also affect the direction of immune responses. Although inflammatory factors are mainly secreted locally, such as in the lymph nodes, to stimulate immune cell activation and only a few enter the blood system, such changes in the cytokine levels can also be monitored in the blood. Our cytokine-focused analyses emphasized that both Th1 and Th2 responses could be elevated by NP-RUs. To further determine whether NP-RUs could produce RU specific antibodies after immunization, Balb/c mice were immunized with one of the four treatments (PBS, OPS, NP-RU, and NP-OPS), containing 2.5 μg of sugar for each except the PBS group, on days 1, 15, and 29. Serum was collected on day 39 (10 days after the last immunization) to assess antibodies against *S. flexneri* 2a 301 LPS. ELISA results showed that although all mice in the NP-OPS group elicited a strong IgG response and half of the mice in the OPS group induced a positive response, there was no positive serum in the NP-RU group (Figure 4D). Since the immune dose was determined by the 2.5 μg of polysaccharide used in the OPS, NP-RU, and NP-OPS groups, the protein mass of NP-RU was the highest, which was far higher than that for normal use. Therefore, this result indicated that the RU special antibody response could hardly be induced by NP-RU, suggesting that the short sugar chain modified nanoparticles could be used as a delivery carrier.

### 3.5. Construction of a Flexible Chassis Using Biotinylation of Nanoparticles

**Figure 5:**
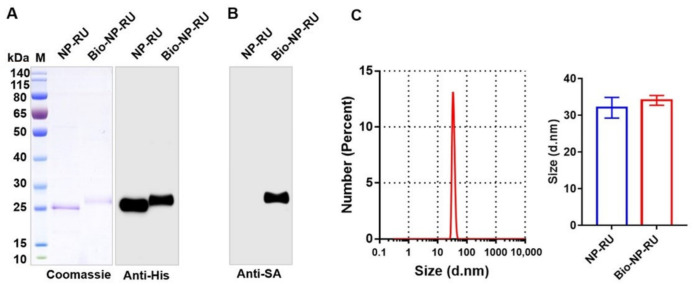
Analysis of biotinylated NP-RUs. NP-RU was biotinylated using EZ-Link NHS-LC-LC-Biotin Reagents (Thermo Fisher, American) and the product was analyzed by Coomassie blue staining and anti-6 × His Western blotting (**A**). (**B**) Biotin on NP-RU was detected through Western blotting using HRP-conjugated streptavidin (Anti-SA). (**C**) DLS analysis of Bio-NP-RU. Each experiment was carried out at least twice.

Encouraged by the clear immunostimulatory effects of NP-RU, we further explored the feasibility of loading various antigens with these particles. As reported, mSA could bind to biotin with a high affinity and we hypothesized that if the NP-RU was biotinylated, antigens containing mSA sequences could be coupled through the interaction between biotin and mSA. Therefore, biotinylated NP-RUs (Bio-NP-RU) was first prepared by using an in vitro biotinylation kit. According to the instruction, the mixture of biotin reagent and NP-RU solution was incubated at 25 °C for 1 h, and then biotinylated nanoparticles were produced by removing unbound biotin via dialysis. Coomassie blue staining and anti-His tagged Western blotting results showed a slight increase in the molecular weight of proteins that were connected to biotin (about 244 Da) (Figure 5A). We used HRP-labeled streptavidin to detect these nanoparticles with or without biotinylation, and further determined that the protein molecular weight migration was caused by biotinylation (Figure 5B). It can be seen from our Western blotting results that the biotinylation efficiency of these nanoparticles practically reached 100%, which meant that there was no need to further remove any unbiotinylated nanoparticles, but only dialysis to remove unbound biotin. DLS results also indicated that Bio-NP-RU remained monodisperse and there was no significant difference in size between NP-RU and Bio-NP-RU mainly due to the small molecular weight of biotin (Figure 5C).

### 3.6. Display of Protein Antigen Types on NP-RU Chassis

**Figure 6:**
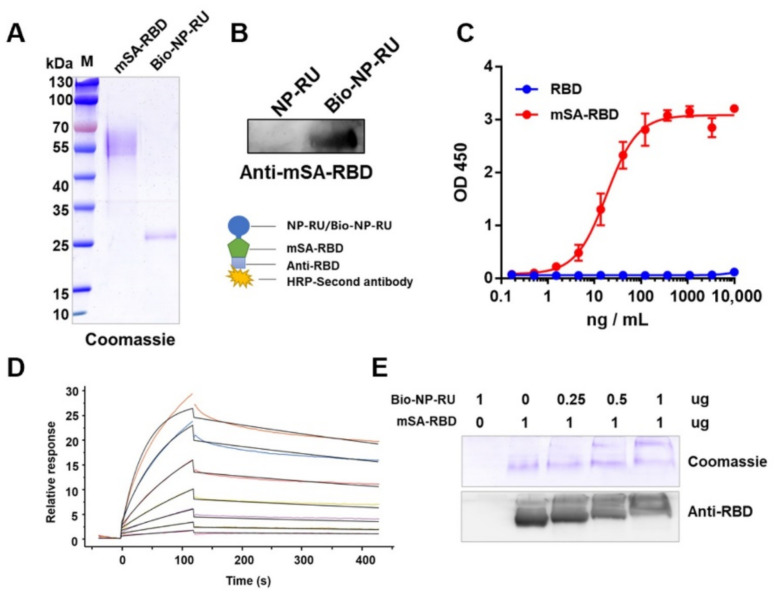
Protein antigen RBD was loaded on Bio-NP-RU through mSA-biotin. (**A**) Coomassie blue staining analysis of purified mSA-RBD and Bio-NP-RU. (**B**) NP-RU and Bio-NP-RU were separated through SDS-PAGE and transferred onto PVDF membranes. The connection between Bio-NP-RU and mSA-RBD was detected through Western blotting, in which mSA-RBD, Anti-RBD, and secondary antibodies were incubated in turn. (**C**) Functional ELISA was performed to measure the binding ability between Bio-NP-RU and mSA-RBD. We coated 96-well plates with Bio-NP-RU at 1 μg/mL (100 μL/well), and RBD and mSA-RBD were gradually diluted from 10 μg/mL. (**D**) The Bio-NP-RU affinity of mSA-RBD was detected by SPR. mSA-RBD was immobilized on chip and Bio-NP-RU was analyzed. Lines with different colors indicated the different concentrations of Bio-NP-RU. (**E**) mSA-RBD was mixed at different proportions with Bio-NP-RUs and incubated at 37 °C for 1 h. The combination between Bio-NP-RU and mSA-RBD was analyzed via Coomassie blue staining and Western blotting after being separated by non-denaturing electrophoresis. Each experiment was carried out at least twice.

Considering the continuing outbreak of SARS-CoV-2 worldwide and the serious threat to the public, we selected its receptor-binding domain (RBD) on the S protein as an antigen for our next evaluation. We constructed a plasmid (pcDNA3.1-mSA-RBD) for expressing mSA-RBD, in which mSA was fused at the N-terminal of RBD. After introducing this into HEK293 cells, expressing the protein and purifying it, mSA-RBD with a purity of greater than 90% was obtained (Figure 6A). Coomassie blue staining results showed a cluster of bands from the purified mSA-RBD with higher molecular weights than anticipated. When mSA-RBD was digested with PNGase F, the molecular weight decreased by over 10 kDa (Supplementary Materials Figure S6), suggesting that mSA-RBD was glycosylated during expression. Since it was found that the RBD purified under the same expression conditions only decreased less than 10 kDa after being digested with PNGase F (Supplementary Materials Figure S7), we speculated that the glycosylation modification also occurred in the mSA sequence. In addition, we analyzed the binding ability of mSA-RBD to ACE2 by functional ELISA and the results showed that there was only a slight decrease in the binding of ACE2 compared with RBD, suggesting that the structure of RBD was not significantly affected by the fusion with mSA (Supplementary Materials Figure S8). After that, two samples, NP-RU and Bio-NP-RU, were separated by SDS-PAGE and transferred to PVDF membranes, and then incubated with mSA-RBD, Anti-RBD and HRP-labeled secondary antibodies in turn. As a result, a positive band was detected in the Bio-NP-RU lane at the corresponding molecular weight (Figure 6B). Then, Bio-NP-RU was immobilized on 96-well plates and the binding ability with mSA-RBD was detected by functional ELISA. A dose-response relationship between mSA-RBD, but not RBD, and Bio-NP-RU was revealed (Figure 6C). These results indicated that mSA-RBD could bind to Bio-NP-RU directly. Furthermore, through surface plasmon resonance, we determined that the affinity between mSA-RBD and Bio-NP-RU (K_d_ = 2.01 nM) was consistent with the results of previous prokaryotic expressions of mSA (Figure 6D). Moreover, we mixed a fixed mass of mSA-RBD with different proportions Bio-NP-RUs at 37 °C for 1 h and then these mixtures were separated by non-denaturing electrophoresis and analyzed using Coomassie blue staining and Western blotting. The results showed that the mSA-RBD bands became weaker and weaker with increasing concentrations of biotinylated NP-RU (Bio-NP-RU) (Figure 6E). Since the size of this nanoparticle was too large to enter the gel, the weakening of the mSA-RBD band was a result of its combination with Bio-NP-RU. Further, DLS results showed that the sample remained monodisperse when loading RBD, and the size was significantly improved (Supplementary Materials Figure S9), indicating a successful loading of antigen.

As known, the interaction between biotin and avidin is one of the strongest non-covalent interactions (K_d_ ~ 10^−15^ M) [55], and there has been a growing interest in exploring this non-covalent interaction in nanoscale drug delivery systems for pharmaceutical agents, including small molecules, proteins, vaccines, monoclonal antibodies, and nucleic acids [56,57,58]. Although the reported affinity of mSA with biotin (K_d_ ~ 10^−9^ M) is lower than that of streptavidin [25,26], the nM level is still conducive to maintaining the coupling state between mSA and biotin in vivo. In addition, compared with streptavidin, the smaller molecular weight of mSA and the characteristics of monomer binding with biotin are more conducive to as a handle for coupling with various functional modulars. Moreover, we prepared mSA-RBD in the eukaryotic expression system to obtain RBD with the correct structure. As expected, mSA was glycosylated during expression. However, the affinity between mSA and biotin was 2.01 nM, which consisted of the data from mSA expressed in prokaryotes, suggesting that glycosylation has little effect on the binding. Therefore, we have expanded the application scope of mSA by proving the feasibility of expressing mSA in the eukaryotic system.

### 3.7. Display of Polysaccharide Antigen Types on the NP-RU Chassis

**Figure 7:**
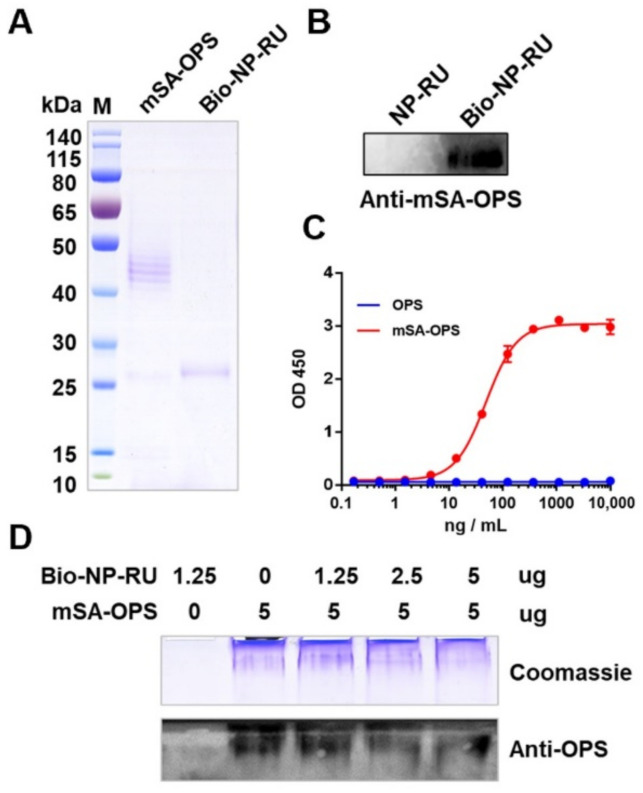
Polysaccharide antigen loaded onto Bio-NP-RU through an mSA connector. (**A**) Coomassie blue staining analysis of purified mSA-OPS and Bio-NP-RU. (**B**) Western blotting was performed to analyze the connection between Bio-NP-RU and mSA-OPS by incubating with mSA-OPS, Anti-OPS, and a secondary antibody in turn. (**C**) Functional ELISA was performed to measure the affinity between Bio-NP-RU and mSA-OPS. We coated 96-well plates with Bio-NP-RU at 1 μg/mL (100 μL/well). OPS and mSA-OPS were diluted from 10 ug/mL. (**D**) mSA-OPS was mixed with different proportions of Bio-NP-RUs and incubated at 37 °C for 1 h. The combination between Bio-NP-RU and mSA-OPS was analyzed via Coomassie blue staining and Western blotting after being separated by non-denaturing electrophoresis. Each experiment was carried out at least twice.

Having confirmed that our strategy could couple protein antigens to the nanoparticle surface, we next studied the application of the binding of other kinds of antigens to these nanoparticles. For bacteria, polysaccharide antigens have strong specificity and are widely used in vaccine development. However, because polysaccharides alone are T cell independent (TI) antigens, they need to be coupled with an appropriate carrier to become T cell dependent (TD) antigens, so that T cells can participate in the process of the immune response [59]. In our study, we attempted to use protein glycosylation modification to realize the efficient coupling between polysaccharide antigens and mSA. Taking *Shigella* polysaccharide antigen as an example, we constructed the expression vector pET-pglL-mSA4573, which co-expressed PglL and an mSA connector, and in which a glycosylation modification sequence (4573) was fused at the C-terminal of mSA. Furthermore, then this vector was introduced into our previously reported host bacteria 301DWP [18], where the O-antigen ligase and virulence plasmid have been deleted from *Shigella flexneri* (2a type 301 strain), and these cells were induced with IPTG. Whole bacterial protein samples were then separated by SDS-PAGE and used for Western blotting. The result indicated that *Shigella* OPS was successfully transferred to mSA with a typical ladder-like band (Supplementary Materials Figure S10). After determining that the purity of mSA-OPS and Bio-NP-RU was greater than 90% by Coomassie blue staining (Figure 7A), further bonding experiments were carried out by ELISA, as described above. The result showed that with the increase in the amount of mSA-OPS, the binding signal gradually increased and finally reached a maximum (Figure 7B), indicating that Bio-NP-RU could also bind with mSA-OPS under natural conditions. Moreover, the results of non-denaturing electrophoresis also showed that the band from mSA-OPS gradually weakened with increasing concentration of Bio-NP-RU (Figure 7C), and DLS results revealed the monodisperse and increased size of Bio-NP-RU loading mSA-OPS (Supplementary Materials Figure S11). These results suggested that these nanoparticles could successfully be loaded with polysaccharide antigens. In addition, the mSA containing glycosylation site sequence could be used as a connector to connect a variety of different biotin proteins and bacterial polysaccharide antigens, which makes this system more widely applicable in its potential.

In the process of vaccine delivery, stable binding between antigen and carrier is an important guarantee for the delivery efficiency and the final protective effect. Although to our knowledge there is no detailed report about this non-covalent interaction in the in vivo environment, many in vitro or animal experiments have indirectly confirmed its great contribution in the process of vaccine action. Zhang et al. developed a multiple antigen-presenting system by coupling biotinylated sugar antigen with rhizavidin, a dimer biotin-binding protein that has the same binding site residues of mSA [26,60], and found that the specific antibody response of the coupled polysaccharide in mice was significantly higher than that of the polysaccharide alone [61]. In addition, when biotinylated cargoes were delivered by carriers fusing monomeric streptavidin, the delivery efficiency, uptake, and transduction in vitro were significantly enhanced [28]. Therefore, the non-covalent binding could play an obvious role in the process of delivery. Moreover, in terms of therapeutic vaccines, Lv et al. developed an anticancer platelet-based biomimetic formulation, in which the non-covalent interaction between the biotinylated nanoparticle and avidin-labeled anti-CD42a antibody could prompt the internalization of the nanoparticles into platelets via the CD42a molecules. Effective therapeutic effects were subsequently demonstrated in nine different mouse models, indicating the great contribution of the non-covalent interaction in biological actions [62]. Besides, a study also showed that mSA2 expressing CAR T cells can target cancer cells coated with biotinylated antibodies through the interaction between mSA2 and biotin, and mediated cancer cell lysis [35], which provided a way for the universal tumor tarting using non-covalent interactions. In our research, we established a proof-of-concept of modular nanovaccine delivery design for the loading of various antigens by the non-covalent binding of mSA and biotin. Thus, the combination strategy was further applied to the preparation of prophylactic vaccines, especially nanoscale polysaccharide conjugate vaccines. Although we have not yet performed the evaluation of vaccine efficacy, the reported results suggest that the binding of antigens with the delivery carrier is reliable, and next, the role of this interaction in vaccine response will be further analyzed through serious animal experiments.

## 4. Conclusions

In our work, we prepared an orthogonal, modular, and highly versatile nanovaccine delivery chassis that could load various antigens (including protein antigens and polysaccharide antigens) using biotin and mSA. We first increased the yield of these nanoparticles by decorating the surface of these particles with short sugar chains via glycosylation modification. Then, we confirmed that this nanoparticle could enhance both Th1 and Th2 immune responses through the analysis of cytokine profiles. Furthermore, we established a proof-of-concept of our modular nanovaccine delivery design for the loading of various antigens by biotin and mSA. After successfully loading the protein antigen (RBD), the fusion expression with mSA on this biotinylated nanoparticle chassis was realized, and we subsequently coupled the polysaccharide antigen to these particles by introducing a designed mSA connector (mSA containing glycosylation modification sequences). It should be emphasized that these nanoparticles still had a high affinity for both protein and polysaccharide antigens, indicating the great potential of this strategy to prepare various types of vaccines. Our approach thus offers an attractive technology for the combination of nanoparticles and antigen cargos. In particular, we have demonstrated here a new efficient strategy for coupling polysaccharide antigens to prepare nanoscale conjugate vaccines.

## Data Availability

The data presented in this study are available on request from the corresponding author.

## Supplementary Materials

The following supporting information can be downloaded at: https://www.mdpi.com/article/10.3390/nano12050734/s1, Table S1: Primers used in this study; Figure S1: Structure diagram of the polysaccharide repeat unit of the *Shigella flexneri* 301 O-polysaccharide; Figure S2: Flow chart of knock out of *rfc* (A) and PCR analysis of mutants in 301DR using primers *rfc*-out-f/r, *rfc*-in-f/r and kan-in-f/r; Figure S3: Flow chart of knock out of *waaI*; Figure S4: Photographs of the Tyndall effect of NP-RU in solution and its size distribution before and after being placed at 37 °C for 48 h; Figure S5: 2 μg of sample was injected subcutaneously in mice and the dynamic change of cytokines IL-22 in the plasma was analyzed using the liquichip method; Figure S6: Coomassie blue staining was used to detect glycosylated mSA-RBD incubated with or without PNGase F; Figure S7: Coomassie blue staining was used to detect glycosylated RBD incubated with or without PNGase F; Figure S8: Functional ELISA was performed to measure the binding capacity of RBD or mSA-RBD produced in mammalian cells to human ACE2; Figure S9: DLS analysis of Bio-NP-RU loading mSA-RBD; Figure S10: Coomassie blue staining and Western blot analysis of the expression of mSA loaded with polysaccharide antigen; Figure S11: DLS analysis of Bio-NP-RU loading mSA-OPS.
