# Supplementary material for: Construction of Orthogonal Modular Proteinaceous Nanovaccine Delivery Vectors Based on mSA-Biotin Binding
Source: Nanomaterials (Basel). 2022 Feb 22;12(5):734. doi: 10.3390/nano12050734 (PMC8911943; doi:10.3390/nano12050734)
Supplement: Supplementary file 1 [file nanomaterials-12-00734-s001.zip › nanomaterials-1580050-supplementary.pdf]

Supplementary Materials

Construction of orthogonal modular proteinaceous nanovaccine delivery vectors based on mSA-biotin binding

Yixin Shi [1,2,†], Chao Pan [2,†], Kangfeng Wang [2,3], Peng Sun [2],Jun Wu [2], Yan Liu [2], Yange Sun [2], Yan Guo [2], Ying Lu [1,*], Li Zhu [2,*] and Hengliang Wang [2,*]

[1] College of Food Science and Technology, Shanghai Ocean University, 999 Hucheng Huan Road, LinGang New City, Shanghai 201306, China; syxsyx0906@163.com(Y.S.)

[2] State Key Laboratory of Pathogen and Biosecurity, Beijing Institute of Biotechnology, 20 Dongdajie Street, Fengtai District, Beijing 100071, China; panchaosunnny@163.com(C.P.); sunpeng990718@163.com(P.S.); junwu1969@163.com(J.W.); liuyanAMS@163.com(Y.L.); sunyg1995@163.com(Y.S.); yanguobrilliant@163.com(Y.G.)

[3] College of Life Science, Hebei University, 180 Wusi East Road, Baoding 071002, China; wangkf1220@126.com(K.W.)

* Correspondence: y-lu@shou.edu.cn(Y.L.); jewly54@126.com(L.Z.); wanghl@bmi.ac.cn(H.W.)

† Yixin Shi and Chao Pan contributed equally to this work and share first authorship.

Table S1. Primers used in this study.

Primer	Sequence (5'→3')
rfc-up-f	CGGGATCCGGTGGAAGATTACTGG
rfc-up-r	GCGTCGACAAAACTGATAATCGCACCG
rfc-down-f	CCAAGCTTTAGGCGTTGTAGTTTT
rfc-down-r	CCCTCGAGATACATAAGCCCCTCA
rfc-out-f	ATAGCTAATGCCTTAGCTGATGATAGCTTTG
rfc-out-r	AGGTTGCTGCACATATAAAACCAACAATGTT
rfc-in-f	GTGCTTGCGATATTATTGAC
rfc-in-r	AACCAAATCCTCTTCCAAAC
waaI-up-f	CGGGATCCGGGTATGGGAAGAATCAAG
waaI-up-r	GCGTCGACTCAGAAATGCTACGGTGT
waaI-down-f	CCAAGCTTTGGACCTTTAGACAATCAA
waaI-down-r	CCCTCGAGATGTTAGGAAGCATACCG
waaI-out-f	CTACAGATGCTGGCGAATA
waaI-out-r	TCACTACAGTTGGGATGG
waaI-in-f	TGGGTGGGATTACAAGGT
waaI-in-r	CCAATGACTAACACGGAAA
kan-in-f	GGCACAACAGACAATCGGCT
kan-in-r	CCTTCCCGCTTCAGTGACAA

α-D-Glc
1
↓
4
→2)-α-L-Rha-(1→2)-α-L-Rha-(1→3)-α-L-Rha-(1→3)-β-D-Glc*p*NAc-(1→

Figure S1. Structure diagram of the polysaccharide repeat unit of the Shigella flexneri 301 O-polysaccharide.

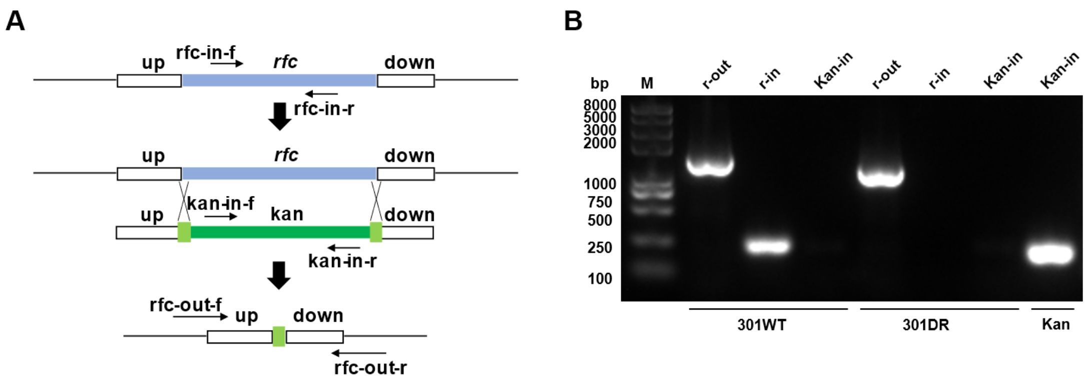

Figure S2. Flow chart of knock out of *rfc* (A) and PCR analysis of mutants in 301DR using primers rfc-out-f/r, rfc-in-f/r and kan-in-f/r.

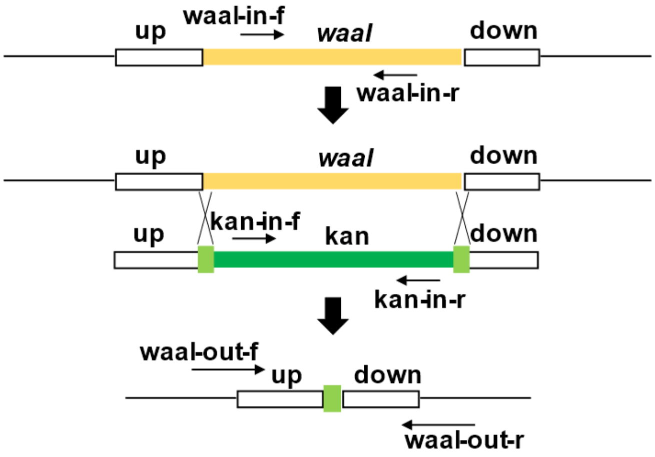

Figure S3. Flow chart of knock out of *waaI*.

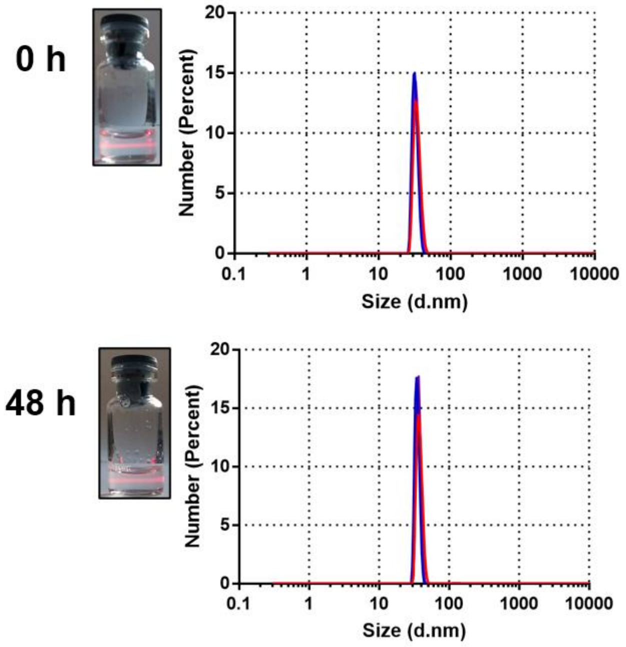

Figure S4. Photographs of the Tyndall effect of NP-RU in solution and its size distribution before and after being placed 37°C for 48 h.

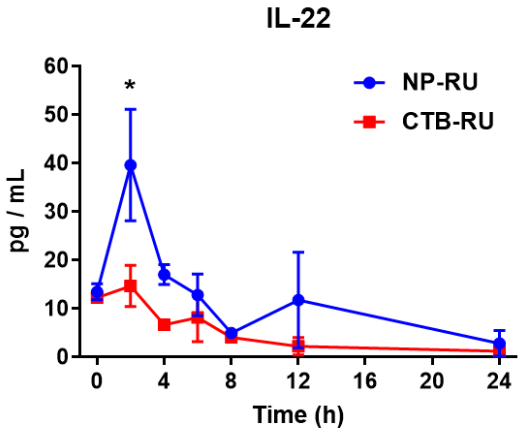

Figure S5. 2 μg of sample was injected subcutaneously in mice and the dynamic change of cytokines IL-22 in the plasma was analyzed using the liquichip method.

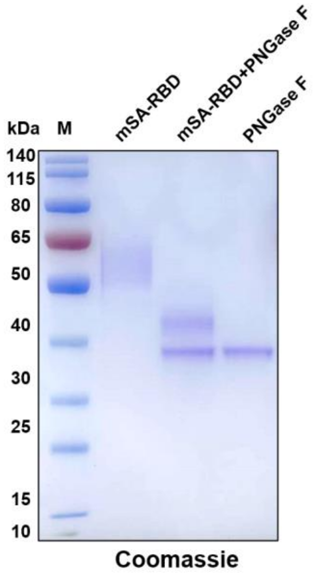

Figure S6. Coomassie blue staining was used to detect glycosylated mSA-RBD incubated with or without PNGase F.

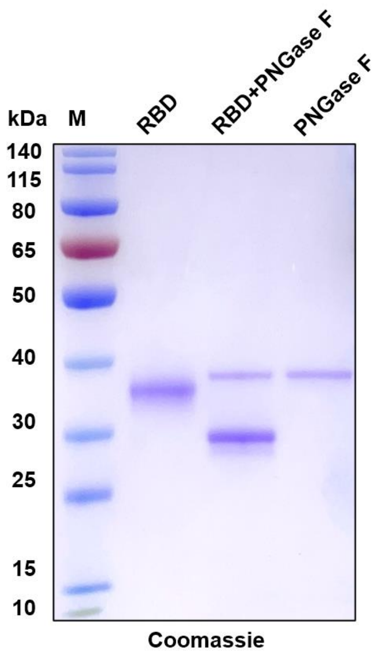

Figure S7. Coomassie blue staining was used to detect glycosylated RBD incubated with or without PNGase F.

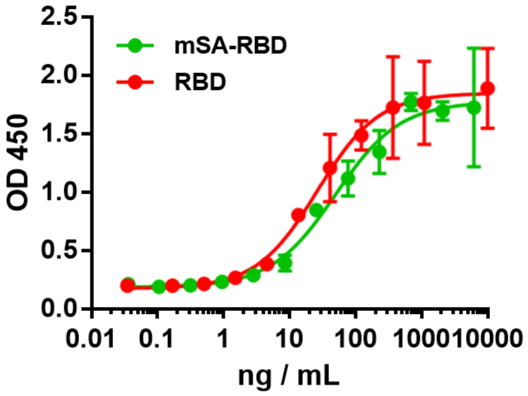

Figure S8. Functional ELISA was performed to measure the binding capacity of RBD or mSA-RBD produced in mammalian cells to human ACE2. The 96-well plate was coated at 2 μg / mL (100 μL / well). RBD and mSA-RBD was diluted from 10 ug / mL as indicated.

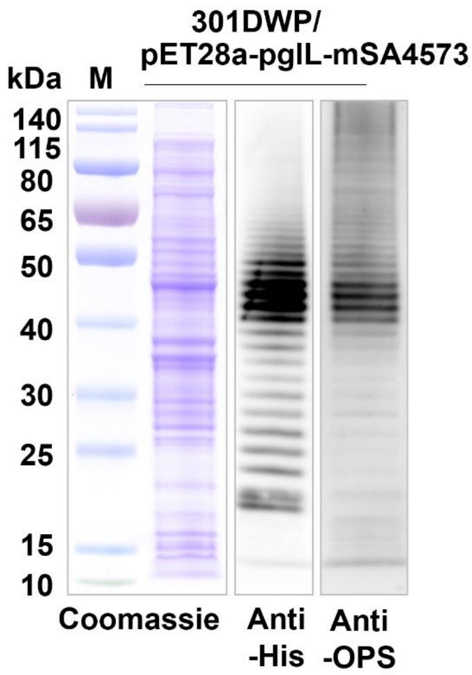

Figure S9. Coomassie blue staining and Western blot analysis of the expression of mSA loaded with polysaccharide antigen.
